# Curbing the appetites and restoring the capacity for satisfaction: The impact of GLP-1 agonists on the reward circuitry

**DOI:** 10.1016/j.nsa.2025.105512

**Published:** 2025-03-03

**Authors:** Anna Julia Krupa

**Affiliations:** Department of Affective Disorders, Jagiellonian University Medical College, Kopernika St. 21a, 31-501, Krakow, Poland

**Keywords:** GLP-1 analog, Semaglutide, Appetite, Reward, Positive affect

In the work titled “GLP-1 receptor agonist semaglutide reduces appetite while increasing dopamine reward signaling,” Kooji et al. examined how the GLP-1 receptor agonist semaglutide impacts dopamine signaling in the ventral tegmental area (VTA) during anticipatory and consummatory phases of food reward processing in mice using a Pavlovian sucrose paradigm. They found that semaglutide reduced appetite and did not alter VTA dopamine activity during the reward-seeking (cue) phase but enhanced VTA dopamine signaling during the reward-collection (consummatory) phase.

Globally, diseases linked to impaired reward processing, such as obesity, mood, and substance use disorders, wreak havoc on public health. Therefore, it is crucial to understand its underlying pathophysiology and mechanisms of effective treatments. The VTA is central to reward signaling, sending dopamine projections to the prefrontal cortex (PFC) via the mesocortical pathway and to the ventral striatum via the mesolimbic pathway (especially nucleus accumbens, NAc), and regulates different domains of reward-seeking behavior, such as reinforcement learning and motivation to work for reward (Gruber et al., 2023). Systemically applied GLP-1 agonists have been clearly shown to modulate the activity of various reward-related structures in the central nervous system - such as the hypothalamus, nucleus tractus solitarius (NTS), and lateral septum - thereby reducing food intake as well as alcohol, cocaine, or nicotine-seeking behaviors (Eren-Yazicioglu et al., 2021; Merkel et al., 2024). However, their direct impact on VTA and its underlying mechanism remains to be clarified. The work of Kooij and colleagues confirmed the impact of GLP-1 agonists on the VTA (Kooij et al., 2024). Most recent findings indicated that the pathway from peripherally administered GLP-1 to decreased reward seeking is via NTS, which projects GLP-1 secreting neurons to VTA-located GABA neurons, and that these, in turn, decrease dopamine activity in VTA (Merkel et al., 2024).

Moreover, the commented work indicated that, while semaglutide did not affect reward-seeking, it enhanced VTA activity during reward consumption (Kooij et al., 2024). It should be mentioned here that the study was performed in food-restricted, lean mice to ensure a high motivation for sucrose rewards. These results, while interesting, should be validated in additional models that better represent clinical populations who either benefit from GLP-1 agonist treatment or could be future targets. Since reward processing is significantly influenced by insulin resistance and obesity, their findings should be validated not only in models of obesity, depression, and substance use but also in models that reflect their frequent co-occurrence in clinical settings. Furthermore, finding that semaglutide had no effect on reward-seeking but increased VTA activity during reward consumption may have implications for treating disorders associated with reward system dysfunction. One could hypothesize that in individuals with substance use, GLP-1 agonists would curb the excessive VTA activity in the cue phase of reward processing (craving, anticipation of relief upon consumption) and promote abstinence (Eren-Yazicioglu et al., 2021). On the other hand, in depression, GLP-1 agonists could promote VTA activity in the consummatory phase of reward processing and reduce anhedonia (Siwek et al., 2024).

Regarding the subjects with obesity, it seems that reward circuitry is altered in individuals with excessive weight, which might predispose some subjects to a perpetual cycle of overeating. Indeed, a human study has shown that 1) BMI is positively correlated with reward anticipation towards palatable food cues and negatively correlated with appraisal of food consumption, 2) GLP-1 agonist exenatide reverses these effects resulting in lower levels of anticipatory and higher consummatory reward, which leads to lower food intake 3) the activity of different reward system regions varies between obese subjects with type 2 diabetes mellitus (T2DM) and those with normoglycemia (van Bloemendaal et al., 2015). However, the alterations of reward system functioning in obesity are still to be discerned, as they are not solely due to higher body mass index (BMI) but appear to be linked to insulin and other hunger/satiety hormones signaling (Gruber et al., 2023) and are altered by comorbid T2DM (van Bloemendaal et al., 2015).

Notably, GLP-1 agonists were initially introduced as antidiabetic and antiobesity drugs but are increasingly popular, gaining increased public and social media coverage and demands that exceed their availability even despite regulations on their access (Strumila et al., 2024). GLP-1 agonists have many benefits on cardiovascular and renal health (lower risk of major cardiovascular events, reduction of cardiovascular risk factors such as body weight or lipid profile, slower progression of chronic kidney disease)(Tempia Valenta et al., 2024). Due to our growing understanding of the close connections between hunger/satiety signaling and the functioning of the reward system (Gruber et al., 2023; Krupa et al., 2024; Siwek et al., 2024), GLP-1 agonists are gaining significant interest in psychiatry. They are being explored in psychiatric populations struggling with altered functioning of the reward system and deficits in positive affect, such as patients with mood disorders, substance use disorders, schizophrenia, and Parkinson's disease (Tempia Valenta et al., 2024).

A recent systematic review showed that GLP-1 agonists alleviate depression and promote cognition in mood disorders, reduce the severity of alcohol and other substance use disorders, decrease binge eating behaviors, and improve cognitive functioning in schizophrenia (Tempia Valenta et al., 2024). On the other hand, warnings were issued on the depression and suicidality risk related to GLP-1 agonists, raising concerns regarding their safety. Recent analyses indicate that the relationship between GLP-1 agonists and suicidality is statistically significant but not causal. The findings are not easy to discern, showing a higher risk of depression and suicidal thoughts in individuals receiving GLP-1 agonists but a lower risk of suicide attempts and completed suicides (McIntyre et al., 2025; Strumila et al., 2024). Thus, caution should be exercised in patients treated with GLP-1 agonists because their rapid and substantial impact on weight changes might cause psychological and physiological stress and have an ambiguous influence on depressive and suicidal symptoms in some individuals. Then again, if managed carefully, GLP-1 agonist treatment might serve as an effective tool to combat depression and lower the risk of suicide (McIntyre et al., 2025; Strumila et al., 2024).

In sum, the work by Kooij and colleagues brings us a step closer to understanding the complexities of GLP-1's impact on reward processing. Hopefully, future studies will further their efforts to provide a more coherent understanding of both the physiological and psychological impact of GLP-1 in clinical populations. These are direct interventions that could effectively modulate impaired reward processing.

## Funding

This research received no specific grant from funding agencies in the public, commercial, or not-for-profit sectors.

## Declaration of competing interest

The authors declare the following financial interests/personal relationships which may be considered as potential competing interests: Anna Julia Krupa reports a relationship with 10.13039/501100006546Angelini Pharma Poland that includes: consulting or advisory, funding grants, speaking and lecture fees, and travel reimbursement. Anna Julia Krupa reports a relationship with Lundbeck Poland that includes: travel reimbursement. Anna Julia Krupa reports a relationship with Swixx Biopharma Sp. z o.o. that includes: travel reimbursement. Anna Julia Krupa reports a relationship with 10.13039/501100003358Gedeon Richter Poland that includes: travel reimbursement. Anna Julia Krupa reports a relationship with Sandoz Polska Sp z oo that includes: travel reimbursement. If there are other authors, they declare that they have no known competing financial interests or personal relationships that could have appeared to influence the work reported in this paper.
